# Combined tumor necrosis factor-α (−308 G/A) and tumor necrosis factor-β (+ 252 A/G) nucleotide polymorphisms and chronicity in Egyptian children with immune thrombocytopenia

**DOI:** 10.1007/s12185-023-03551-9

**Published:** 2023-02-18

**Authors:** Mona El-Ghamrawy, Nesrine El-Gharbawi, Gehan Shahin, Alaa Abdelhady, Rasha Sayed, Nehal Diaa, Irene Bishai

**Affiliations:** 1 Pediatric Hematology & BMT Unit, Pediatrics Department, Faculty of Medicine, Cairo University, Cairo, Egypt; 2 Clinical and Chemical Pathology Department, Faculty of Medicine, Cairo University, Cairo, Egypt

**Keywords:** Chronic ITP, Egyptian, Genotyping, Cytokines, TNF-α, TNF-β, Polymorphisms, Restriction fragment length polymorphism

## Abstract

**Background:**

Primary immune thrombocytopenia (ITP) is a common autoimmune disorder. Secretion of TNF-α, TNF-β and IFN-γ plays a major role in the pathogenesis of ITP.

**Objective:**

This cross-sectional study aimed to detect TNF-α (−308 G/A) and TNF-β (+ 252 A/G) gene polymorphism in a cohort of Egyptian children with chronic ITP (cITP) to clarify their possible association with progression to chronic disease.

**Methods:**

The study included 80 Egyptian cITP patients and 100 unrelated age- and sex-matched controls. Genotyping was performed using polymerase chain reaction–restriction fragment length polymorphism (PCR–RFLP).

**Results:**

Patients with TNF-α homozygous (A/A) genotype had significantly higher mean age, longer disease duration and lower platelet counts (*p* values 0.005, 0.024 and 0.008, respectively). TNF-α wild (G/G) genotype was significantly more frequent among responders (*p* = 0.049). Complete response was more frequent among wild (A/A) TNF-β genotype patients (*p* = 0.011), and platelet count was significantly lower among homozygous (G/G) genotype (*p* = 0.018) patients. Combined polymorphisms were strongly associated with susceptibility to chronic ITP.

**Conclusion:**

Homozygosity in either gene might contribute to a worse course of disease, increased severity and poor response to therapy. Patients expressing combined polymorphisms are more prone to progression to chronic disease, severe thrombocytopenia and longer disease duration.

**Supplementary Information:**

The online version contains supplementary material available at 10.1007/s12185-023-03551-9.

## Introduction

Primary immune thrombocytopenia (ITP) is an autoimmune disease associated with reduced peripheral circulating platelets count. The reduced peripheral blood platelets count is a result of a combination of premature platelet destruction [1] and a relative inadequacy of platelet production [2]. It is characterized by the presence of antibody-sensitized platelets in the reticuloendothelial system; such autoantibodies are specific for platelet membrane glycoproteins, such as GPIIb/IIIa, GPIb/IX and GPIa/IIa [3, 4]**.** Better understanding of the underlying pathophysiology of ITP allowed recent recognition of other mechanisms that are clearly involved. These include T cell-mediated apoptosis of megakaryocytes, inhibition of platelet production and T cell-mediated destruction of platelets [5]. Experts have set a platelet count of less than 100 × 10^9^/L with no clear initiating and/or underlying causes as the diagnostic threshold for primary ITP [6]**.** In children with chronic ITP (cITP), the disease often has a gradual onset occurring in older children lasting for more than 12 months with rare incidence of spontaneous remission and commonly requires therapy [6, 7]**.**

Several genes are involved in immune system regulation like cytokine genes, FcγR gene, cytotoxic T lymphocyte-associated protein-4 (CTLA-4) gene, and human leukocyte antigen (HLA) gene [8, 9]**.** A disruption in the balance of Th1, Th2 and Th17 with secretion of proinflammatory cytokines plays a major role in the pathogenesis of ITP [10, 11]**.** This includes dysfunction of regulatory T cells, increased T lymphocyte-mediated cytotoxicity and elevated Th17 cells, on one hand [11], and release of proinflammatory cytokines (TNF-α and TNF-β) on the other. Overproduction of TNF-α, TNF-β and IFN-γ explains the Th1-related autoreactive cellular immune responses in ITP [12]**.** Such proinflammatory cytokines may play a fundamental role in the pathogenesis of chronic course of the disease, which might be the base for future specific immunomodulatory therapies for cITP in children. This cross-sectional study aimed at detecting TNF-α (-308 G/A) and TNF-β (+ 252 A/G) gene polymorphism in a cohort of Egyptian children with cITP to clarify their possible association with chronic evolution of the disease.

## Patients and methods

The present work included 80 Egyptian patients diagnosed to have cITP (defined as immune thrombocytopenia that lasted longer than 12 months duration) followed up at the Pediatric Hematology Outpatient Clinic of Cairo University Children Hospital, Faculty of Medicine, Cairo University. One hundred age- and sex-matched unrelated Egyptian healthy children were included as a control group. An informed consent was willingly obtained from all parents prior to the study. The study protocol was approved by the Research Committee of Faculty of Medicine, Cairo University on 21/12/2016.

The patient group included 44 males (55%); their age ranged from 2 to 14 years with a mean of 7.08 ± 3.64 years. whereas the age- and sex-matched Egyptian healthy unrelated control group (n = 100) had a mean age of 7.64 ± 3.52 years with 56% being males. Patients with secondary causes of ITP such as HCV, SLE, underlying malignancy or drug-induced thrombocytopenia were excluded. None of the patients had positive ANA, anti-HCV antibody or anticardiolipin antibody. All patients had normal levels of C3 and C4. A thorough review of medical records and direct examination of all subjects were performed.

Complete response (CR) was defined as any platelet count of at least 100 × 10^9^/L and response (R) was defined as any platelet count between 30 and 100 × 10^9^/L and at least doubling of the baseline count. On the other hand, no response (NR) was defined as any platelet count lower than 30 × 10^9^/L or less than doubling of the baseline count [13]**.** Refractory patients were those fulfilling two criteria: first, those who failed splenectomy or relapsed thereafter; second, those who exhibit severe ITP or have a risk of bleeding and require therapy.

Clinical and hematological characteristics for cITP patients are attached as a supplementary file.

### Sampling and specimen collection

Ten ml blood samples were collected under aseptic conditions by means of clean venipuncture in a vacutainer containing EDTA, of which 2 ml was for performing CBC, 5 ml for laboratory screening and 3 ml blood for PCR–RFLP analysis of TNF-α (-308G/A) and TNF-β (+ 252A/G) gene polymorphisms.

### DNA extraction and amplification from peripheral blood leucocytes

One hundred microliters (100 μl) of the sample (whole blood) was worked upon for extracting DNA using the Quick-gDNA™ MiniPrep kit (catalog No: D3024, USA). Amplification was performed through repetitive cycles of DNA denaturation, primer annealing and extension by DNA polymerase using MyTaq™ Red Mix (Bioline, Australia). The primers for TNF-α and TNF-β genes were provided by (Biosearch Technologies, USA). The restriction enzyme (RE) used is NcoI-HF (Biolabs, New England, no. 0061510). TNF-α (-308 G/A) and TNF-β (+ 252 A/G) gene polymorphisms among cITP patients and control group were interpreted according to the number of bands and bp of each genotype (Figures S1 and S2 shown in supplementary file).

## For TNF-α gene polymorphism:

- Wild G/G genotype gives two bands at 87 bp and 20 bp.
- Heterozygous G/A genotype gives three bands at 107, 87 and 20 bp.
- Homozygous A/A genotype gives a single band at 107 bp.

## For TNF-β gene polymorphism:

- Wild A/A genotype gives a single band at 782 bp.
- Heterozygous A/G genotype gives three bands at 782, 586 bp and 196 bp.
- Homozygous G/G genotype gives two bands at 586 bp and 196 bp.

### Statistical analysis

Data were coded and entered using the statistical package SPSS version 23. Data were expressed using mean, standard deviation, median, minimum and maximum for quantitative variables, and frequencies (number of cases) and relative frequencies (percentages) for categorical variables. Comparisons between quantitative variables were done using the non-parametric Kruskal–Wallis and Mann–Whitney tests. For comparing categorical data, Chi-square (χ2) test was performed. Exact test was used instead when the expected frequency was less than 5. Genotype and allele frequencies were compared between the disease and the control groups using Chi-square tests. Odds ratio (OR) with 95% confidence intervals was calculated. *p* values less than 0.05 were considered statistically significant.

## Results

In this study, TNF-α genotyping revealed that the frequency of wild G/G, heterozygous G/A and homozygous A/A genotypes among cITP patients were 81.2%, 15.0% and 3.8% versus 79.0%, 20.0% and 1.0% among the control group, respectively, with no statistically significant difference between both groups. TNF-β genotyping revealed that the frequency of wild A/A, heterozygous A/G and homozygous G/G genotypes among cITP patients was 55%, 40% and 5% versus 60%, 28% and 12% in the control group, respectively (Table 1).

**Table 1:** Comparison between chronic ITP patients and control group with regard to TNF-α and TNF-β genes polymorphism and allele frequencies

Item	cITP group *n* = 80	Control group *n* = 100	*p* value*
TNF-α gene (− 308 G/A) polymorphism (No., %)			
Wild GG genotype	65 (81.2%)	79 (79.0%)	Reference
Heterozygous GA genotype	12 (15.0%)	20 (20.0%)	0.4
Homozygous AA genotype	3 (3.8%)	1 (1.0%)	0.3
G allele	142 (88.8%)	178 (89.0%)	Reference
A allele	18 (11.3%)	22 (11.0%)	0.9
TNF-β gene polymorphism (+ 252 A/G) (No., %)			
Wild AA genotype	44 (55.0%)	60 (60.0%)	Reference
Heterozygous AG genotype	32 (40.0%)	28 (28.0%)	0.2
Homozygous GG genotype	4 (5.0%)	12 (12.0%)	0.2
G allele	40 (25.0%)	52 (26.0%)	0.8
A allele	120 (75.0%)	148 (74.0%)	Reference

*cITP* chronic ITP

*p value < 0.05 is considered significant

### TNF-α gene genotypes in terms of demographic, clinical and laboratory data

**Fig. 1:**
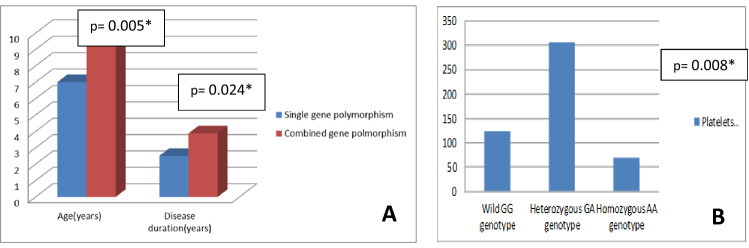
Comparison between TNF-α gene genotypes with regard to **A** age and disease duration, **B** platelet count (× 10^9^/L). *p value < 0.05 is considered significant

Comparison between wild (G/G), heterozygous (G/A) and homozygous (A/A) genotypes of TNF-α gene showed a statistically significant difference with regard to age, disease duration and platelet count (*p* values 0.005, 0.024 and 0.008, respectively); patients with homozygous (A/A) genotype showed significantly higher mean age, longer disease duration and lower platelet count (Fig. 1). There was no statistically significant difference between TNF-α gene genotypes regarding sex or other clinical/ laboratory characteristics. Notably, a comparison of responsive (CR + R) cITP patients (n = 55) versus unresponsive (NR + refractory) patients (n = 25) with regard to both studied genotypes and allele frequencies showed that the TNF-α wild (G/G) genotype was significantly more frequent among responsive patients, while heterozygous (G/A) and homozygous (A/A) genotypes were more frequent among unresponsive patients (p = 0.049) [data not shown].

### TNF-β genotypes in terms of demographic, clinical and laboratory data

**Fig. 2:**
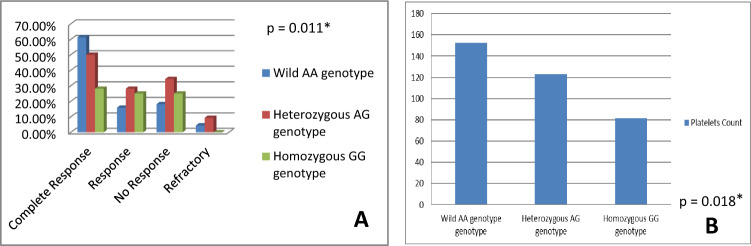
Comparison between TNF-β gene genotypes with regard to **A** treatment response, **B** platelet count (× 10^9^/L). *p value < 0.05 is considered significant

Comparison of TNF-β genotypes revealed a statistically significant difference with regard to treatment response and platelet count; complete response was more frequent among the wild (A/A) genotype (*p* value 0.011) and platelet count was significantly lower among homozygous (G/G) genotype (*p* value 0.018) (Fig. 2). No other statistically significant differences were detected regarding demographic data and other clinical and laboratory characteristics.

Comparison between TNF-α and TNF-β genotypes among cITP patients with regard to demographic clinical and hematological data are shown in a supplementary file.

### Single gene polymorphism vs. combined genes polymorphism for cITP with regard to demographic and clinical data

**Fig. 3:**
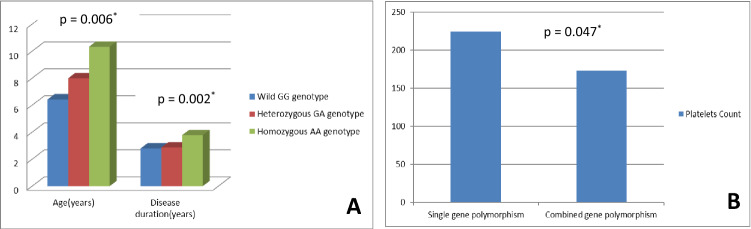
Comparison between cITP patients with single gene polymorphism versus those with combined genes polymorphism with regard to: **A** age and disease duration, **B** platelets count (× 10^9^/L). *p value < 0.05 is considered significant

Comparison was done between patients exhibiting polymorphism of either TNF-α or TNF-β genes (a single gene polymorphism) and patients exhibiting polymorphisms of both TNF-α and TNF-β genes (combined gene polymorphism). Combined gene polymorphisms revealed statistically significant difference regarding age, disease duration and platelet count (*p* values 0.006, 0.002 and 0.047, respectively) [Fig. 3]; age and disease duration were significantly higher, and platelet count significantly lower among patients with combined gene polymorphisms. No other significant differences were detected regarding other demographics, clinical data or treatment response.

Comparing single and combined TNF-α and TNF-β gene polymorphisms in cITP patients and the control group with regard to risk of cITP development, a statistically significant difference between the two groups was noted (p = 0.015). Calculated risk estimation revealed that combined gene polymorphisms conferred threefold increased risk of development of cITP (OR = 3.491, 95%CI: 1.235–9.869) [Table 2].

**Table 2:** Comparison between chronic ITP patients and control group with regard to single and combined gene polymorphisms as a risk for chronic ITP

Item	cITP group *n* = 80	Control group *n* = 100	OR	95% CI	*p* value
Single gene polymorphism *n* = 25	25 (65.8%)	47 (87.0%)	Reference		
Combined gene polymorphisms *n* = 13	13 (34.2%)	7 (13.0%)	3.5	1.2–9.9	0.015*

*cITP* chronic ITP

*p value < 0.05 is considered significant

## Discussion

It has been suggested that susceptibility to autoimmune diseases may be directly related to polymorphisms in the TNF genes, such as TNF-α (-308G/A) gene [14–16] and TNF-β (+ 252A/G) [4, 17]**.** However, findings from various studies were conflicting. This cross-sectional study aimed at detecting TNF-α (-308 G/A) and TNF-β (+ 252 A/G) gene polymorphisms in a cohort of Egyptian children with cITP to clarify their possible association with chronic evolution of the disease.

In this study, TNF-α (308 G/A) genotyping showed no significant difference in the frequencies of genotypes between cITP patients and controls. This is in concordance with the findings by other studies [4, 17–19]**.** In contrast, Pehlivan et al. (2011) and Mokhtar et al. (2016) reported higher frequency of A/G genotype in patients with ITP [14, 20]**.**

Our work revealed no significant difference in the frequencies of TNF-β (+ 252 A/G) genotypes between patients and controls. This is in agreement with other reports [18, 21]**.** However, two studies reported higher frequency of A allele in ITP patients than controls [4, 17]**.**

Discrepancies in findings between studies might be attributed to variations in sample size and dissimilarity in patients' selection including disease stage or ethnic differences in some populations.

Our observation that patients with homozygous TNF-α (A/A) or TNF-β (G/G) genotypes or combined genetic polymorphisms had significantly lower platelet counts suggests that these genetic polymorphisms could be implicated in the severity of thrombocytopenia and might affect disease severity. In addition, patients with homozygous TNF-α (A/A) genotype or combined gene polymorphisms were significantly older and had longer disease duration, affecting disease duration and course.

In our study, TNF-α wild (G/G) genotype was significantly more frequent among responsive cITP patients, while heterozygous (G/A) and homozygous (A/A) genotypes were more frequent among unresponsive patients. In agreement, one study showed that the A allele of TNF-α (308G/A) was more frequent in patients with unresponsive ITP [4]**.** Another study reported significantly higher TNF-α A/G phenotype in steroid-refractory and splenectomized cases at the end of the first year than in steroid-responsive and remission cases [14]**.** Thus, TNF-α (308 G/A) gene polymorphism may contribute to therapy resistance in cITP.

Our study showed that the probability of having a complete response to treatment was highest among the wild A/A genotype of TNF-β (+ 252 A/G) and the least among the heterozygous A/G genotype. In contrast, two studies did not show any effect of TNF-β (+ 252 A/G) gene polymorphisms on treatment response [4, 21]**.** A possible explanation may be differences in patients’ sample size, age group, disease stage and ethnic or geographic background.

Both studied genes are present within the MHC locus on chromosome 6p. The TNF-α gene is located in the class III region of the MHC on chromosome 6p21.33. TNF-α −308G/A polymorphism has been previously found to increase TNF-α transcription by six- to eightfold and has been associated with increased TNF-α production and development of several autoimmune diseases including ITP. The TNF-β gene is adjacent to the TNF-α gene within a 7-kb locus in the MHC. The TNF-β (+ 252G/A) polymorphism is located in the first intron and it correlates with the level of TNF-β protein production by lymphocytes [22, 23]. Individuals having TNF-β (+ 252) G allele are high producers of TNF-β, which is produced by activated T cells and is involved in the maturation and activation of B cells and associated with the risk of development of breast, gastric and autoimmune diseases including ITP [17, 18, 22, 24, 25]**.** Thus, TNF-β (+ 252G/A) polymorphism affects the expression of both genes and the concentration of TNF-α and TNF-β proteins in plasma [26]**.** Our study showed that neither the variant genotypes of TNF-α GA/AA and its minor A allele nor variant genotypes of TNF-β AG/GG, and its minor G allele alone are risk factors for the susceptibility to cITP in pediatric Egyptian patients. However, patients expressing combined gene polymorphisms are more prone to the development of chronicity; combined gene polymorphisms conferred threefold increased risk of development of cITP. Few studies suggested that the homozygous G/G genotype of TNF-β may be associated with increased susceptibility to ITP or increased risk for chronic ITP development [17, 18]**.** In contrast, Morgan et al., 2018 showed no increased risk for developing ITP was associated with any TNF-β allele/genotype [21]. Two studies suggested that TNF-α as gene of susceptibility to ITP [14, 15]**.** However, Okulu et al., 2011 reported no association between TNF-α gene polymorphism and the risk of developing ITP or its clinical progress [19]**.** A systematic meta-analysis of eight high-quality case–control studies, including 947 patients and 1911 controls, revealed that TNF-α -308G/A under the dominant model (AA + AG versus GG) might significantly increase ITP risk in Caucasians, however, no statistically significant association was observed in overall and Asian populations, revealing that allele A carrier (AA + AG) of rs1800629 might increase predisposition to ITP in Caucasians. This was explained by the higher frequency of A allele in Caucasian population and relatively small sample size in cases and control among Asian populations. Besides, TNF-α −308G/A polymorphism was reported to affect gene transcription by increasing TNF-α production and regulating cell proliferation and differentiation, leading to potentially exacerbate the outcomes of ITP [27]**.** Discrepancies between studies could be a reflection of several factors, such as sample size, patients’ selection criteria, and genetic heterogeneity of various ethnic populations or different age groups or that these two gene polymorphisms are in linkage disequilibrium with other neighboring genes. It also may be attributed to differences in disease pathogenesis and disease progression between different age groups.

Our results indicate that homozygous genotypes of either TNF-α or TNF-β genes might contribute to worsening of disease course, aggravating disease severity and having poor response to therapy. A novel finding in our study is that patients expressing combined studied gene polymorphisms are more prone for disease chronicity and severe thrombocytopenia with longer disease duration. Thus, the expression of TNF-α and TNF-β gene polymorphisms seems to play an important role in the pathogenesis of childhood cITP and may contribute to modification of the disease course and response to treatment. However, multiple other genetic and environmental factors play a role in the pathogenesis of ITP. The possible contribution of TNF-α (308 G/A) gene polymorphism to therapy resistance is worth further studying.

Further research needs to be extended including larger cohorts of patients and longer periods of follow-up to allow for the development of novel therapeutic targets and hence a better disease outcome.

## Conclusion

A novel finding in our present study is the significant association between combined polymorphisms of both TNF-α and TNF-β genes and susceptibility to developing chronicity of ITP in Egyptian children. Patients expressing combined gene polymorphisms have a more severe disease form and prolonged disease duration. Screening for TNF-α and TNF-β gene polymorphism might be of value for ITP patients to predict disease severity and response to treatment.

## Supplementary Information

Below is the link to the electronic supplementary material.

Supplementary file1 (DOCX 755 KB)
