# Supplementary material for: Combined tumor necrosis factor-α (−308 G/A) and tumor necrosis factor-β (+ 252 A/G) nucleotide polymorphisms and chronicity in Egyptian children with immune thrombocytopenia
Source: Int J Hematol. 2023 Feb 18;117(6):856–62. doi: 10.1007/s12185-023-03551-9 (PMC10225363; doi:10.1007/s12185-023-03551-9)
Supplement: Supplementary file 1 — Supplementary file1 (DOCX 755 KB) [file 12185_2023_3551_MOESM1_ESM.docx]

Supplementary File


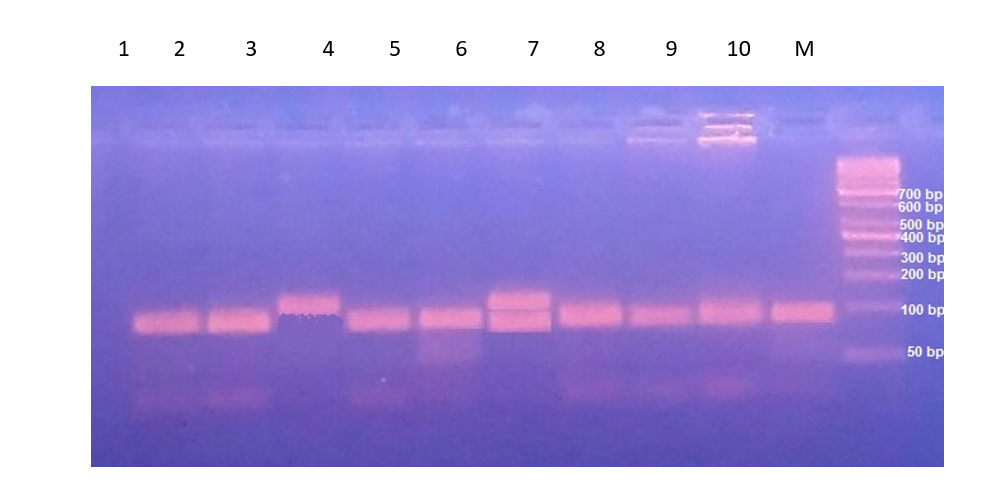


**Figure S1: Agarose gel electrophoresis of TNF-α gene (-308 G/A) PCR products**

**Enzyme digestion products of TNF-α (-308G/A) using NcoI restriction enzyme showing:**

**Lanes 1,2,4,5,7,8,9,10: Wild GG genotype showing bands at 87 bp and 20 bp.**

**Lane 6: Heterozygous AG genotype showing bands at 107, 87 and 20 bp.**

**Lane 3: Homozygous AA genotype showing single band at 107 bp.**

**M= DNA marker**


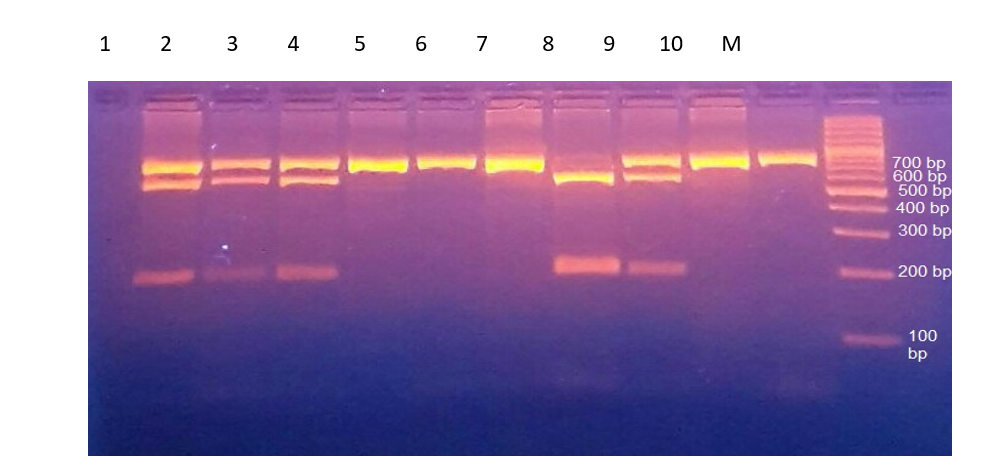


**Figure S2: Agarose gel electrophoresis of TNF-β gene (+252 A/G) PCR products.**

Enzyme digestion products of TNF-β (+252G/A) using NcoI restriction enzyme showing:

**Lanes** 4,5,6,9,10: Wild AA genotype showing band at 782 bp.

**Lane** 7: Homozygous GG genotype showing bands at 586 bp and 196 bp.

**Lanes** 1,2,3,8: Heterozygous AG genotype showing bands at 782, 586 bp and 196 bp.

**M**= DNA marker.

**Clinical and hematological characteristics of chronic ITP patients**

| **Item** | **cITP**  **(n=80)** |
| --- | --- |
| **Disease duration (years)**  **Did** | |
| Range | 1-9 |
| Mean ± SD | 2.95±3.64 |
| **Bleeding at initial diagnosis (No., %)** | |
| Subcutaneous or mucosal bleeding | 59(73.75%) |
| GIT or genitourinary bleeding | 12(15%) |
| Severe or life-threatening bleeding | 0(0%) |
| **Splenectomy (No., %)** | 11(13.75%) |
| **Treatment response (No., %)** | |
| Complete response | 38(47.5%) |
| Response | 17(21.25%) |
| No response | 20 (25%) |
| Refractory | 5 (6.25%) |
| **Hematological parameters (Mean ±SD, Range)** | |
| Hb (g/dl) | 10.91±1.48 (5.90-13.40) |
| TLC (x10^9^/L) | 8.72±2.55 (4.50-15.50) |
| Platelets (x10^9^/L) | 122.61±105.95 (1-455) |

**cITP: chronic immune thrombocytopenia; GIT: gastrointestinal;** **Hb: hemoglobin; TLC: total leucocytic count**

**Comparison between TNF-α genotypes among chronic ITP patients as regards demographic, clinical and hematological data**

| **Parameter** | **Wild genotype GG n=65** | **Heterozygous genotype GA n=12** | **Homozygous genotype AA n=3** | **p-value** |
| --- | --- | --- | --- | --- |
| **Age (years)** | | | | |
| Range | 2-14 | 3-11 | 6-14 | **0.005^*^** |
| Mean ± SD | 6.43±3.39 | 8±4.36 | 10.33±3.26 |  |
| **Sex (No.,%)** | | | | |
| Male | 36(55.4%) | 6(50%) | 2(66.7%) | 0.901 |
| Female | 29(44.6%) | 6(50%) | 1(33.3%) |  |
| **Disease duration (years)** | | | | |
| Range | 1-9 | 1.6-5 | 2-6 | **0.024^*^** |
| Mean ± SD | 2.80±1.89 | 2.87±1.86 | 3.78±1.04 |  |
| **Treatment response (No.,%)** | | | | |
| Complete response | 31(47.7%) | 4(33.3%) | 3(100%) | 0.147 |
| Response | 16(24.6%) | 1(8.3%) | 0(0%) | 0.427 |
| No response | 15(23.1%) | 5(41.7%) | 0(0%) | 0.337 |
| Refractory | 3(4.6%) | 2(16.7%) | 0(0%) | 0.318 |
| **Hematological parameters (Mean ±SD)** | | | | |
| Hb (g/dl) | 10.95±1.51 | 10.58±1.46 | 11.40±1.13 | 0.624 |
| TLC (x10^9^/L) | 8.68±2.65 | 8.76±1.90 | 9.40±3.45 | 0.767 |
| Platelets (x10^9^/L) | 123.83 ±104.64 | 306.67±28.87 | 70±67 | **0.008^*^** |

**Hb: hemoglobin; TLC: total leucocytic count; *P value <0.05 is considered significant.**

**Comparison between TNF-β genotypes among chronic ITP patients as regards demographic, clinical and hematological data.**

| **Parameter** | **Wild genotype AA n=44** | **Heterozygous genotype AG n=32** | **Homozygous genotype GG n=4** | **p-value** |
| --- | --- | --- | --- | --- |
| **Age (years)** | | | | |
| Range | 2-14 | 2-14 | 4-10 | 0.688 |
| Mean ± SD | 6.80±3.74 | 7.47±3.67 | 7±2.58 |  |
| **Sex (No.,%)** | | | | |
| Male | 23(52.3%) | 18(56.2%) | 3(75%) | 0.752 |
| Female | 21(47.7%) | 14(43.8%) | 1(25%) |  |
| **Disease duration (years)** | | | | |
| Range | 1-9 | 1.20-6 | 1.40-5 | 0.572 |
| Mean ± SD | 2.88±2.01 | 3.05±1.55 | 2.95±1.60 |  |
| **Treatment response (No., %)** | | | | |
| Complete response | 27(61.4%) | 9(28.1%) | 2(50%) | **0.011^*^** |
| Response | 7(15.9%) | 9(28.1%) | 1(25%) | 0.410 |
| No response | 8(18.2%) | 11(34.4%) | 1(25%) | 0.216 |
| Refractory | 2(4.5%) | 3(9.4%) | 0(0.0%) | 0.727 |
| **Hematological parameters (Mean ±SD)** | | | | |
| Hb (g/dl) | 11.01±1.31 | 10.68±1.73 | 11.68±0.96 | 0.541 |
| TLC (x10^9^/L) | 8.65±2.59 | 8.52±2.50 | 11.10±1.54 | 0.106 |
| Platelets (x10^9^/L) | 152.68±115.37 | 123±118.24 | 81.22±75.74 | **0.018^*^** |

**Hb: hemoglobin; TLC: total leucocytic count; *P value <0.05 is considered significant.**
